# Cognitive-behavioral therapy for binge eating disorder in adolescents: study protocol for a randomized controlled trial

**DOI:** 10.1186/1745-6215-14-312

**Published:** 2013-09-25

**Authors:** Anja Hilbert

**Affiliations:** 1 Department of Medical Psychology and Medical Sociology, Integrated Research and Treatment Center AdiposityDiseases, University of Leipzig Medical Center, Philipp-Rosenthal-Strasse 27, Leipzig, 04103 Germany

**Keywords:** Adolescents, Binge eating disorder, Cognitive-behavioral therapy, Randomized-controlled trial

## Abstract

**Background:**

Binge eating disorder is a prevalent adolescent disorder, associated with increased eating disorder and general psychopathology as well as an increased risk for overweight and obesity. As opposed to binge eating disorder in adults, there is a lack of validated psychological treatments for this condition in adolescents. The goal of this research project is therefore to determine the efficacy of age-adapted cognitive-behavioral therapy in adolescents with binge eating disorder – the gold standard treatment for adults with binge eating disorder.

**Methods/design:**

In a single-center efficacy trial, 60 12- to 20-year-old adolescents meeting diagnostic criteria of binge eating disorder (full-syndrome or subthreshold) according to the Diagnostic and Statistical Manual of Mental Disorders 4^th^ or 5^th^ Edition, will be centrally randomized to 4 months of cognitive-behavioral therapy (n = 30) or a waiting-list control condition (n = 30). Using an observer-blind design, patients are assessed at baseline, mid-treatment, post-treatment, and at 6- and 12-month follow-ups after the end of treatment. In 20 individual outpatient sessions, cognitive-behavioral therapy for adolescents focuses on eating behavior, body image, and stress; parents receive psychoeducation on these topics. Primary endpoint is the number of episodes with binge eating over the previous 28 days at post-treatment using a state-of-the art clinical interview. Secondary outcome measures address the specific eating disorder psychopathology, general psychopathology, mental comorbidity, self-esteem, quality of life, and body weight.

**Discussion:**

This trial will allow us to determine the short- and long-term efficacy of cognitive-behavioral therapy in adolescent binge eating disorder, to determine cost-effectiveness, and to identify predictors of treatment outcome. Evidence will be gathered regarding whether this treatment will help to prevent excessive weight gain. If efficacy can be demonstrated, the results from this trial will enhance availability of evidence-based treatment of adolescent binge eating disorder.

**Trial registration:**

German Clinical Trials Register: DRKS00000542

## Background

Binge eating disorder (BED), a provisional category in the Diagnostic and Statistical Manual of Mental Disorders Fourth Edition (DSM-IV-TR) [1], was approved in the fifth revision DSM-5 [2] as its own diagnostic category of eating disorder. BED is characterized by recurrent binge eating that occurs, in contrast to the binge eating in bulimia nervosa (BN), in the absence of regular inappropriate compensatory behavior to prevent weight gain (e.g., self-induced vomiting). BED has been identified in 1.6% of 13- to 18-year-old adolescents from the community [3], and in up to 25% of treatment-seeking adolescents [4,5]. However, it is still debated as to whether the DSM diagnostic criteria adequately capture the presentation of BED in children and adolescents. For example, beyond objective binge eating as defined in the DSM criteria of BED (i.e., loss of control over eating a significantly large amount of food) [2], a substantial proportion of adolescents report subjective binge eating that is not part of the DSM diagnostic criteria of BED (i.e., loss of control over eating a subjectively large amount of food), but is psychopathologically relevant [4,6] (both types of binge eating are hereafter referred to as binge eating). In addition, lowered diagnostic thresholds have been proposed for the diagnosis of BED in youth [7,8]. Subthreshold BED was found to be associated with marked impairment and increased risk of developing a full-syndrome presentation over time [9].

Similar to BED in adults, this disorder in adolescence is associated with increased eating disorder psychopathology (e.g., weight concern) and general psychopathology (e.g., depression, anxiety), impaired quality of life, and overweight and obesity [4,10,11]. BED and its associated eating disorder and general psychopathology were found to be longitudinally predicted by binge eating in youth [12-14]. As postulated by maintenance theories of binge eating developed for adults [15,16] and validated for youth [17], binge eating in youth may be maintained through negative affect and difficulties in regulating these mood states [18-20]. Weight-related teasing and a negative body image were closely linked with greater binge eating in youth [21-23]. Further, binge eating emerges in the context of increased impulsivity, irregular meal patterns, and interpersonal problems such as dysfunctional family interactions [24-27]. These results suggest that patterns of non-normative eating behavior, difficulties in regulating negative emotional states, a negative body image, and interpersonal problems are important targets of intervention.

Of great concern are findings that youth with binge eating or BED show increased weight gain and obesity onset over time [14,28-31]. Given the psychological, social, and medical sequelae of obesity [32,33], the risk of tracking the excess body fat and comorbidities into adulthood [34,35], and the associated health-care costs [36], it is of the highest relevance to develop efficacious interventions for the treatment of BED in adolescents to alleviate this disorder’s symptomatology and to prevent excessive weight gain.

Related to the recency of research about BED in adolescence, there is a lack of validated treatments for this age group. For adults with BED, cognitive-behavioral therapy (CBT) is the most well-established psychological treatment, as reflected in systematic reviews [37-39], meta-analysis [40], and clinical guidelines [41], although superiority to other bona fide treatments has not been demonstrated clearly [42]. Efficacy studies of CBT documented substantial reductions in binge eating and associated symptomatology that can be maintained over the long-term [43]. There is also evidence that patients who achieve abstinence from binge eating lose a small but significant amount of weight when compared to those who remain symptomatic [44,45].

Among the few studies evaluating CBT or other psychological treatments for adolescents with recurrent binge eating, CBT was in a pilot randomized-controlled trial (RCT) compared with a delayed treatment control condition. This CBT trial was efficacious in yielding abstinence from recurrent binge eating over a three-month follow-up in 12- to 18-year-old adolescents (n = 26) [46]. In addition, concerns about shape, weight, and eating were significantly reduced. The age-adapted CBT manual comprised eight individual sessions over 3 months on nutritional management, weight control, and body image, and up to four optional sessions on food and mood and interpersonal relationships. Another study focused on an Internet-based mode of treatment delivery: a 16-week Internet-based intervention program using CBT-principles (among other principles) was part of a waiting-list (WL) controlled randomized study. The Internet program was found to be efficacious in facilitating weight loss or weight maintenance, and reducing recurrent binge eating and shape and weight concerns, in overweight adolescents over a 5-month follow-up period (n = 105) [47]. In addition, a small-scale (n = 38) randomized-controlled pilot study demonstrated feasibility of 12-week group interpersonal psychotherapy in 12- to 17-year-old adolescent girls at risk for overweight, with about half of the patients suffering from binge eating [48]. The results also suggested that interpersonal psychotherapy reduced binge eating at 3-month follow-up and led to better weight maintenance than a standard health education group at 9-month follow-up. Further evidence is available on CBT or CBT-based guided self-help for adolescents with BN or with mixed eating disorder diagnoses [49,50], providing support for CBT-based treatment in the related eating disorder of adolescent BN. Thus far, however, confirmatory evidence on the efficacy of psychological treatments for BED in adolescents is lacking, moderators and mediators are unknown, and cost-effectiveness has not been evaluated.

In this context, the aim of the Binge Eating Disorder in Adolescents (BEDA) study is to adapt an established CBT manual for adult BED to adolescent BED, and evaluate its short- and long-term efficacy, relative to a WL control condition in a single-center randomized trial. Additional objectives are to identify outcome predictors (moderators and mediators) and to evaluate cost-effectiveness.

## Methods/design

### Hypotheses

(1) Age-adapted individual CBT will be superior to WL in reducing the number of binge eating episodes in adolescents with BED 4 months following randomization.

(2) Age-adapted individual CBT will be superior to WL in reducing the number of days with binge eating episodes; leading to greater abstinence from binge eating; reducing eating disorder psychopathology and the severity of depressive symptoms; increasing self-esteem and quality of life; and stabilizing body mass index. The therapeutic gains will occur at mid-treatment and will be maintained at 6 and 12 months following CBT.

(3) Age adapted individual CBT will be cost-effective.

### Design, participants, and procedures

#### Study design

**Figure 1:**
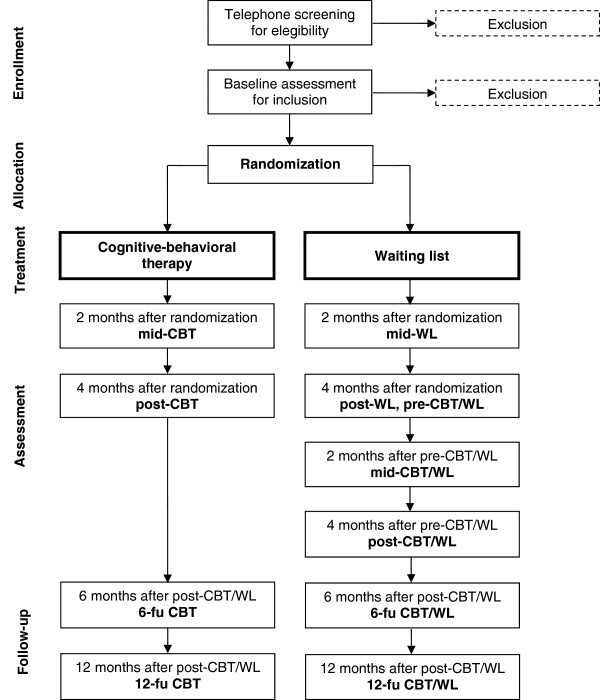
Study design.

BEDA is a single-center, prospective, randomized superiority trial, evaluating the efficacy and cost-effectiveness of CBT (experimental condition) compared to a WL control condition. The study design is depicted in Figure 1. The study period lasts 4 months per patient in the experimental condition (4 months of CBT) and 8 months in the control condition, including 4 months of WL and 4 months of CBT; each patient is offered individual CBT. Using an observer-blind design, patients are assessed at baseline, 2 months after randomization (mid-CBT; mid-WL), 4 months after randomization (post-CBT; post-WL or pre-CBT/WL), and at 6- and 12-months follow-up after the end of treatment (6-fu CBT, 12-fu CBT; 6-fu CBT/WL, 12-fu CBT/WL). The patients in the WL condition are assessed additionally 2 months (mid-CBT/WL) and 4 months (post-CBT/WL) after the end of the waiting period (i.e., after post-WL or pre-CBT/WL).

### Participants

A total of 60 12- to 20-year-old adolescents meeting age-adapted DSM criteria of BED will be randomized to either CBT (n = 30) or WL (n = 30). Inclusion criteria are summarized below. Based on the evidence on classification of BED in youth (see Introduction), age-adapted diagnostic criteria of BED are used in order to determine: BED according to DSM-IV-TR based on objective and/or subjective episodes of binge eating *or* BED according to DSM-5 based on objective and/or subjective episodes of binge eating *or* Subthreshold BED according to DSM-5. To ensure generalization of study results, exclusion criteria are kept to a minimum.

#### Inclusion and exclusion criteria

Inclusion:

• Age 12–20 years

• Diagnosis of BED according to age-adapted DSM criteria;

• BED (DSM-IV-TR): At least 2 days with objective and/or subjective episodes of binge eating per week over the past 6 months; at least 3 out of 5 behavioral indicators; marked distress; absence of regular compensatory behaviors to avoid weight gain; absence of anorexia nervosa and BN;

• BED (DSM-5): At least 1 episode of objective and/xor subjective episodes of binge eating per week over the past 3 months; at least 3 out of 5 behavioral indicators; marked distress; absence of regular compensatory behaviors to avoid weight gain; absence of anorexia nervosa and BN;

• Subthreshold BED (DSM-5). All DSM-5 criteria for BED are met, except the binge eating occurs on average less than once a week and for less than 3 months.

• Written informed consent of parent and adolescent for adolescents ages <18 years, written informed consent of adolescents at ages ≥18 years.

Exclusion:

• Current BN;

• Current substance abuse;

• Current suicidal ideation;

• Psychotic disorder;

• Bipolar disorder;

• Serious unstable medical problems or conditions (e.g., type 1 diabetes mellitus or thyroid problems);

• Current intake of antipsychotic or weight-affecting drugs;

• Current psychotherapy;

• Inpatient psychiatric treatment within the past 3 months prior to screening;

• Current conservative weight loss treatment;

• Female adolescents in pregnancy or lactation;

• Lack of compliance with major study procedures;

• Current participation in another treatment trial.

### Ethical approval

The study was approved by the Ethical Committee at the University of Leipzig Medical Center. Written informed consent of at least one parent and informed consent of the adolescent are obtained for adolescents <18 years after the study has been fully explained. For adolescents ≥18 years, written informed consent of the adolescent only is required. Patients and their parents can withdraw at any point without any penalty. The trial will be conducted in accordance to the guidelines for Good Clinical Practice [51]. All persons participating in the conduct of the trial commit themselves to the Declaration of Helsinki (Version Somerset West 1996) [52], as well as all pertinent national laws and the International Conference on Harmonization guidelines for Good Clinical Practice and CPMP/ICH/135/95 (Note for Guidance on Good Clinical Practice) [53].

### Recruitment

The ongoing study is conducted from September 2011 at the Outpatient Unit of the Integrated Research and Treatment Center (IFB) AdiposityDiseases at University of Leipzig Medical Center, Leipzig, Germany. Recruitment strategies include population-based recruitment (e.g., information letters to households with children in the targeted age range, website http://www.ess-stress.de), school-based recruitment (e.g., educational classes, screening), and recruitment in clinical settings (e.g., IFB Outpatient Unit, behavioral weight loss facilities).

### Procedures

Adolescents are screened by telephone for eligibility in the study. Eligible adolescents and at least one parent are invited to the IFB Outpatient Unit for a diagnostic visit. At the outset of this visit, informed consent is gathered in a written format as described. At baseline assessment, inclusion and exclusion criteria are evaluated using the clinical interviews described below. Additionally, sociodemographic, anamnestic, anthropometric, and clinical data, and health economic parameters, are obtained using interviews, self-report-questionnaires, and objective measurement as described below. After baseline assessment, included patients are centrally randomized into the CBT or WL arm. Following randomization into the CBT arm, patients are offered treatment over 4 months. Following randomization into the WL arm, patients are invited to CBT over 4 months, after a waiting-list period of 4 months.

### Intervention

#### Experimental intervention – Cognitive-behavioral therapy (CBT)

An age-appropriate CBT manual was developed in order to target the above-mentioned psychopathology and maintenance factors of BED in adolescents. This adapted manual was based upon the existing evidence-supported adult manual “Binge eating and obesity: Manual for the treatment of binge eating disorder” by Hilbert and Tuschen-Caffier [54]. This manual for adult BED was piloted in an RCT [55] and is currently used in a multicenter RCT comparing it to Internet-based guided self-help [56]. Recovery and clinically significant improvement rates of the pilot data lay within the range of those determined by other clinical trials evaluating the efficacy of CBT in the treatment of BED [40].

The age-adapted manual for adolescents with BED differs from the adult manual in several aspects: greater focus on less complex, motivating behavioral exercises; decreased focus on cognitive interventions; concentration on age-specific maintenance factors (e.g., difficulties in identity development) including familial factors (e.g., familial eating patterns, family conflict); and a greater focus on enhancing an autonomous motivation for treatment, since adolescents may not necessarily be motivated for treatment and/or treatment may be sought by third parties (e.g., parents).

All adolescent patients are guaranteed confidentiality regarding their parents unless they provide information that meets criteria for mandated reporting. Although family-based treatment is efficacious in adolescent BN [57], parental involvement in adolescent treatment for bulimic eating disorders may decrease compliance [46,50]; thus, treatment is individually focused on adolescents. Parents receive standardized information letters about BED, general intervention topics, and recommendations for daily routine on a monthly basis.

The age-adapted CBT manual includes the following phases: (1) initial treatment phase for motivational enhancement; (2) intensive treatment phase including modules on eating behavior, body image, and stress; and (3) self-management phase for relapse prevention. Therapeutic phases, goals, and techniques are depicted in Table 1.

**Table 1:** Therapeutic phases, goals, and techniques

	**Therapeutic goals**	**Techniques**
	**Initial treatment phase (Sessions 1 – 3)**	
**Initial contact and cognitive preparation**	- To establish a positive therapeutic alliance	- Exploration
	- To enhance motivation	- Information on disorder and treatment
	- To plan therapy	- Goal setting
		- Self-monitoring of eating behavior
		- Self-management and self-reinforcement
	**Intensive treatment phase (Sessions 4 – 18)**	
**Eating behavior module “My Nutrition”**	- To establish a healthy eating behavior	- Information on nutrition and physical activity
		- Self-monitoring of eating behavior and physical activity
		- Goal setting
		- Stimulus and response control
		- Cue exposure
		- Eating hedonics exercises
		- Self-management and self-reinforcement
		- Cognitive restructuring
		- Homework
**Body image module “My Body”**	- To establish a positive body image and weight maintenance	- Information on body image and weight-related stigmatization
		- Body image exposure
		- Self-monitoring of negative body talk
		- Self-monitoring of physical activity
		- Shaping of regular physical activity
		- Body hedonics exercises
		- Self-management and self-reinforcement
		- Cognitive restructuring
		- Homework
**Stress module “My Emotions”**	- To identify and treat related psychopathology (e.g., emotion regulation difficulties, stress management problems, interpersonal conflict, social competence deficits, identity problems)	- Behavioral analysis
		- Information
		- Goal setting
		- Stimulus and response control
		- Role play
		- Problem solving
		- Exposure
		- Self-management and self-reinforcement
		- Cognitive restructuring
		- Homework
	**Self-management phase (Sessions 19 – 20)**	
	- To stabilize progress	- Review of progress
	- To prevent relapse	- Goal setting regarding future work
		- Identification of situations and signs of risk for relapse and delineation of an emergency plan
		- Self-management and self-reinforcement

The age-adapted CBT with the adolescent lasts 4 months and comprises 20 individual face-to-face sessions (50 min) with the adolescent. Within the first month of treatment, two sessions per week are scheduled (sessions 1–8), and in months 2 to 4 one session per week is scheduled (sessions 9–20). CBT treatment is provided by at least three clinical psychologists with advanced training in behavior therapy and a specific training in CBT of BED. Treatment fidelity is ensured through a specific training in the CBT manual and regular supervision, which will also prevent drift in treatment delivery. Patients are allocated to therapists according to their availability, and each therapist will see eight or more patients. Treatment fidelity and therapist adherence will be established using continuous adherence controls based on audio documentation of the psychotherapeutic sessions by two independent, trained raters using five (25% of sessions) randomly-selected tapes per patient. The rating system is specific for CBT of eating disorders, and was developed and evaluated in an independent clinical study of CBT for adults with BED [Brauhardt et al.; unpublished data].

#### Control intervention – waiting list (WL)

Half of the patients will be randomly assigned to a WL control condition. All WL patients are guaranteed individual CBT after a waiting period of 4 months (equal to the duration of CBT). The WL patients are instructed not to seek any other medical or psychological treatment for BED or obesity during the WL period without informing the project leader.

### Measures

#### Primary and secondary outcomes

The primary outcome measure is the number of binge eating episodes over the previous 28 days 4 months after randomization (post-CBT, post-WL), which is a standard measure within treatment trials of adult BED [40]. The number of binge eating episodes is assessed with the German version of the Eating Disorder Examination (EDE) [58-60], a validated semi-structured interview regarded as the gold standard for the diagnosis and psychopathological assessment of eating disorders. If necessary, age-adapted language is used from the child version of the EDE [61,62]. For adult BED, it is common to determine the difference in the number of episodes or days with objective binge eating between baseline and follow-up and test for a difference between a treatment and control group [40]. In adolescents, this measure has severe disadvantages since the large variance in the number of episodes with binge eating (i.e., objective and objective binge eating [47,48]) can result in imbalances between randomized arms (e.g., [47]). Abstinence rates, another typical measure in treatment trials for adult BED [40], have the disadvantage that patients who reduce their binge eating substantially, but not completely, are treated as failures. Hence, we chose to consider the ratio of the number of binge eating episodes at 4 months after randomization to the number at baseline as primary outcome criterion.

Secondary outcome measures include the number of binge eating episodes 2 months after randomization (mid-CBT, mid-WL), and maintenance of treatment success regarding the number of binge eating episodes at 6 and 12 months following CBT (6-fu CBT, 12-fu CBT). Additional secondary outcomes are also measured at 2 and 4 months after randomization and at 6 and 12 months following CBT: the number of days with binge eating; abstinence (i.e., zero binge eating episodes) from binge eating; eating disorder psychopathology (all determined through the EDE); additional aspects of non-normative eating behavior as determined through the Dutch Eating Behavior Questionnaire [63,64]; the severity of depressive symptoms [65,66]; self-esteem [67,68]; quality of life [69,70], and body mass index (BMI, kg/m^2^). Body weight and height is objectively measured through calibrated instruments. Psychiatric comorbidity is determined per structured clinical interview K-DIPS [71] 6 and 12 months following CBT.

We chose these outcome measures because they exhibit good psychometric properties in the respective age group, are well-established in German, and are used in international research studies. The structured clinical interviews EDE and K-DIPS are conducted by trained raters. All raters are blind to study hypotheses and randomization, and have no therapeutic relationship with the patients. They underwent extensive training for conducting the interviews and receive ongoing supervision for standardized administration (drift prevention).

#### Cost effectiveness

Health resource utilization and loss of productivity is assessed using the Client Sociodemographic and Service Receipt Inventory [72], adapted for the purposes of this study. The self-report questionnaire is administered at all assessment time points specified above.

#### Predictor variables

Potential predictor variables, assessed at baseline, consist of outcome variables, sociodemographic and anamnestic variables, and patient motivation and expectation ratings assessed through visual analogue scales. The predictor (moderator) variables will be selected based on the literature or on our theoretical understanding of the mode of action of the treatment.

#### Process variables

For exploratory identification of mediators, process measures are obtained by the patient every week of treatment. These consist of a selection of items of the self-report form of the EDE, the Eating Disorder Examination-Questionnaire [73-75].

### Methodological aspects

#### Power analysis

RCTs analyzing the reduction of binge eating episodes by interpersonal psychotherapy [48], an Internet-based CBT program [47], and adolescent-specific CBT [46] provide rough estimates for baseline situations and treatment effects. In these studies, variances in the number of binge eating episodes are large and abstinence rates vary considerably. Simulation of treatment effects that was repeated 5,000 times demonstrated that a power of 95% can be reached with n = 27 individuals per group using a *t*-test (α = 0.05). With an assumed overall dropout rate of 10% of study patients (range: 0 to 11.5%) [47,48], 30 patients should be recruited per study group. While assuming this dropout rate, every effort is made to retain as many participants as possible throughout the period of the study, including providing information to participants on the relevance and necessity of the study, the use of continuity forms locating participants throughout the study period, and use of incentives for follow-up assessments.

#### Randomization

Adolescents who meet the respective criteria and who give informed consent (and for adolescents aged <18 years whose parents give informed consent) are randomized. To ensure concealment of allocation, the randomization is centrally performed by the Coordination Center for Clinical Trials of the University of Leipzig. Randomization is computer-assisted, using Pocock’s minimization algorithm [76]. The randomization is stratified by sex and age (12 to 15 and 16 to 20 years). The allocation ratio between the two arms of the study is 1:1.

#### Blinding

Outcome assessments are performed by independent raters who have no therapeutic relationship with the patients and are blind to study hypotheses and randomization. Blinding of patients and treatment is not possible in this trial, as both therapists and patients know the study arm from the particular modes of delivery.

#### Data analytic plan

The primary outcome “reduction in the number of binge eating episodes over the previous 28 days 4 months after randomization” will be investigated with analysis of covariance using the ratio as the dependent variable (the number of binge eating episodes at 4 months following randomization divided by the number at baseline), treatment group as factor and the inverse number of binge eating episodes at baseline as a covariate based on the full analysis set [77]. The full analysis set includes all randomized patients, regardless of whether or not they provide data at follow-up. Further, the analysis of the number of binge eating episodes will be performed in the per protocol set to evaluate the treatment effect for patients with good protocol adherence. In addition, the maintenance of treatment effects regarding the number of binge eating episodes will be analyzed in the framework of random coefficients modeling. Random coefficients modeling is state of the art in the analysis of eating disorder treatment effects, as it can incorporate heterogeneity of effects (by treatment moderator) and is tolerant of missing data.

Secondary outcome measures will be analyzed in an exploratory manner and will be evaluated by means of parametric or non-parametric tests, depending on the given scale level and type of distribution of the observed variables. Maintenance of treatment effects will be evaluated by random coefficient models. For all outcomes, effect sizes will be estimated and presented with 95% confidence intervals. Predictors of treatment outcome (moderators and mediators) will be identified in an exploratory manner using multivariable analyses.

#### Monitoring and data management

The trial is performed in cooperation with the Coordination Center for Clinical Trials of the University of Leipzig, which is responsible for monitoring and data management. Data are monitored for completeness, consistency, and plausibility. Errors in data entry are determined in a step-wise procedure, examining all data of five patients and, depending on error rates, examining all data in up to an additional 25% of the patients. Data quality is ensured through plausibility checks (e.g., examination of ranges). Post-treatment data will be released only after study completion (i.e., after termination of the 12-months follow-up). No interim analyses are planned.

The study data will be reported in accordance with the extended Consolidated Standards of Reporting Trials guidelines for non-pharmacological treatment studies [78].

#### Safety aspects

Adverse events are all unwanted medical events (e.g., emerging or aggravating symptoms) occurring throughout the trial whether or not they have a causal association with the trial. Adverse events will be documented at every assessment and at every week of treatment throughout the trial. They are rated regarding their severity: serious adverse events are those having led to death, that are life-threatening, make inpatient treatment necessary, lead to sustained harm, or cause birth defects or deformities. Serious adverse events include mental or physical decompensations that indicate a need for hospitalization (e.g., acute suicidality). Adverse events are recorded through a self-report assessment of somatic symptoms [79,80] at every assessment and an unstandardized reporting of adverse events every week during treatment. In addition, adverse events are assessed systematically at post-treatment using a self-report scale for the assessment of negative effects of psychotherapy [INEP, “Inventar zur Erfassung Negativer Effekte von Psychotherapie” Ladwig et al.; unpublished manuscript].

An independent Data Monitoring and Safety Committee (DMSC) was established to meet once per year for independent study supervision. The DMSC is composed of three researchers with expertise in the area of the study. The type of information monitored includes patient recruitment, number of dropouts, and all adverse events including study withdrawals. Any serious adverse event will be immediately reported to the Ethical Committee at the University of Leipzig Medical Center and will be forwarded to the DMSC. The DMSC receives recruitment and retention updates on a regular basis.

## Discussion

Recent evidence indicates that a substantial proportion of adolescents suffer from BED and associated psychopathology. Additionally, they are at risk of excessive weight gain, overweight, and obesity, with increased risk of associated medical sequelae. As the research interest in adolescent BED has been growing only recently, there is a lack of evidence-based psychological treatments for BED in this age group. One randomized pilot study demonstrated feasibility and suggested potential efficacy of a brief individual CBT for binge eating and associated psychopathology [46]; another randomized clinical trial showed that an Internet-based program based on CBT principles (among other principles) was efficacious in facilitating weight loss or weight maintenance, and reducing binge eating and shape and weight concerns in overweight adolescents with recurrent binge eating [47]. Both trials included mixed populations of recurrent binge eaters: compensatory behaviors [47] and/or BN [46] were not excluded, and binge eating frequently lay below the diagnostic threshold of BED [46]. Both trials suggested maintenance of effects over a short-term follow-up period of three to five months. Since CBT is the most well established psychological treatment of adult BED, an examination of the short-term and long-term efficacy of individual CBT specific for adolescent BED is a logical next step of research.

In this study, an evidence-supported CBT manual for adult BED was adapted for adolescents and is subjected to a confirmatory efficacy test comparing individual CBT to a WL control condition in a randomized psychotherapy trial with a follow-up period of one year. An extension of this long-term follow-up is planned, but will depend on organizational requirements after the end of the funding period. If efficacy can be demonstrated, the results from this trial will enhance availability and foster dissemination of evidence-based treatment of adolescent BED. Beyond the effects on BED and associated psychopathology, evidence will be gathered to determine if this treatment will also help to prevent excessive weight gain. The study will further allow for the identification of individuals for whom (moderators) CBT will work and allow for clarification of which mechanism (mediators) changes will be entailed. Such results would advance decisions for matching adolescents with BED into CBT, and may inform technical aspects of intervention conduct.

## Trial status

This study is ongoing and will continue until April 2015.

## Abbreviations

BED: Binge eating Disorder; BEDA: Binge eating disorder in adolescents; BN: Bulimia nervosa; CBT: Cognitive-behavioral therapy; DMSC: Data Safety and Monitoring Committee; DSM-IV-TR: Diagnostic and Statistical Manual of Mental Disorders Fourth Edition Text Revision; DSM-5: Diagnostic and Statistical Manual of Mental Disorders Fifth Edition; EDE: Eating Disorder Examination; IFB: Integrated Research and Treatment Center AdiposityDiseases; K-DIPS: Structured clinical interview Diagnostisches Interview bei Psychischen Störungen des Kindes- und Jugendalters; RCT: Randomized-controlled trial; WL: Waiting list.

## Competing interests

The author declares that she has no competing interests.

## Authors’ information

AH, PhD, is a clinical psychologist, with a concentration on the psychological treatment of binge eating disorder and obesity in adults, adolescents, and children. She is Professor of Behavioral Medicine and Psychological Director of the Outpatient Unit of the Integrated Research and Treatment Center AdiposityDiseases at the Leipzig University Medical Center, Leipzig, Germany. She serves as the President of German Eating Disorders Society.
